# Surface modification of carbon nanotubes with copper oxide nanoparticles for heat transfer enhancement of nanofluids

**DOI:** 10.1039/c7ra10406e

**Published:** 2018-01-08

**Authors:** Abdallah D. Manasrah, Ismail W. Almanassra, Nedal N. Marei, Usamah A. Al-Mubaiyedh, Tahar Laoui, Muataz A. Atieh

**Affiliations:** Department of Chemical and Petroleum Engineering, University of Calgary 2500 University Drive NW Calgary Alberta Canada T2N 1N4; Chemical Engineering Department, King Fahd University of Petroleum & Minerals Dhahran Saudi Arabia; Mechanical Engineering Department, King Fahd University of Petroleum & Minerals Dhahran Saudi Arabia; Qatar Environment and Energy Research Institute, HBKU, Qatar Foundation PO Box 5825 Doha Qatar mhussien@qf.org.qa; Colleague of Science and Engineering, HBKU, Qatar Foundation PO Box 5825 Doha Qatar

## Abstract

Over the last few years, nanoparticles have been used as thermal enhancement agents in many heat transfer based fluids to improve the thermal conductivity of the fluids. Recently, many experiments have been carried out to prepare different types of nanofluids (NFs) showing a tremendous increase in thermal conductivity of the base fluids with the addition of a small amount of nanoparticles. However, little experimental work has been proposed to calculate the flow behaviour and heat transfer of nanofluids and the exact mechanism for the increase in effective thermal conductivity in heat exchangers. This study mainly focuses on the development of nanomaterial composites by incorporating copper oxide nanoparticles (CuO) onto the surfaces of carbon nanotubes (CNTs). The CNT–CuO nanocomposite was used to prepare water-based heat transfer NFs. The morphological surfaces and loading contents of the CNT–CuO nanocomposite were characterized using field emission scanning electron microscopy (FE-SEM), transmission electron microscopy (TEM), X-ray photoelectron spectroscopy (XPS) and thermogravimetric analysis (TGA) while the physical and thermal properties of the water-based nanofluids were characterized using differential scanning calorimetry (DSC), the Mathis TCi system and a viscosity meter for measuring the heat capacity, thermal conductivity and viscosity of the synthesized NFs, respectively. The heat transfer and the pressure drop studies of the NFs were conducted by a horizontal steel tube counter-flow heat exchanger under turbulent flow conditions. The experimental results showed that the developed NFs with different concentrations of modified CNTs (0.01, 0.05 and 0.1 wt%) have yielded a significant increase in specific heat capacity (102% higher than pure water) and thermal conductivity (26% higher than pure water) even at low concentration. The results also revealed that the heat rate of the NF was higher than that of the base liquid (water) and increased with increasing the concentration of nanoparticles. Furthermore, no significant effect of the nanoparticles on the pressure drop of the system was observed.

## Abstract 2

Over the last few years, nanoparticles have been used as thermal enhancement agents in many heat transfer based fluids to improve the thermal conductivity of the fluids.

## Graphical abstract

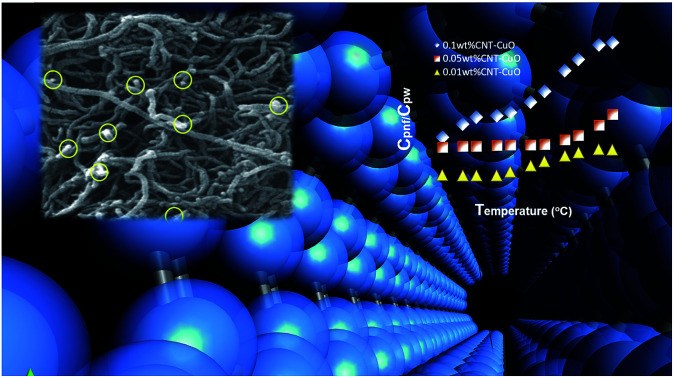

## 1. Introduction

The demand for highly efficient heating and cooling systems is progressively increasing with the expansion of the current technologies and industrial processing applications.^1,2^ The improvement of the heating or cooling systems in any industrial application is one of the main engineering approaches for better energy and cost optimization.^3^ Using an effective heat exchanging system will reduce the processing time and maintain the equipment in a good operation status for a long time.^4^ The heat-transfer fluid is the main effective factor in the heat exchanging system as it directly affects the cost and size of the heat exchanger systems.^5^ Conventional fluids like water and oil are commonly used as a main component of the heat-transfer fluid mixtures.^4,6^ However, due to the low heat capacity and boiling point, these conventional liquids are limiting the ability of the heat exchanger operation. For that reason, extensive research in developing high-performance heat-transfer fluids has been and will continue to be carried out.^7–12^

The nanotechnology, in terms of nanoparticles, has the potential in manufacturing metals and metals oxide with remarkable effects on the thermophysical and transport properties when added to the base fluids.^13–16^ Additionally, the nanoparticles in the fluids showed less clogging abrasion, better dispersion behaviour and the higher surface area which contemplate improving the efficiency of heat-transfer fluids compared with the behaviour of the conventional fluids.^17–19^

The “nanofluid” term for heat exchangers applications has been firstly introduced and described by Choi in 1995 (ref. 4) as “an innovative new class of heat-transfer fluids that can be engineered by suspending nanoparticles in conventional heat transfer fluids”. This nanofluid was reported to be able to improve the thermal conductivity and convective heat transfer performance.^13,14,20,21^ The nanofluids' performance found to be higher than the traditional base fluids (ethylene glycol, water, oils).^22–25^ Since then, many studies have been carried out on the nanofluid enhancement using ultra-dispersed metal oxide nanoparticles into conventional base fluids such as Cu/CuO, Al/Al_2_O_3_, Au, Fe/Fe_3_O_4_, SiO_2_, CeO_2_, and TiO_2_.^13,26–33^ Metal oxide nanoparticles with a size range of 1–100 nm have shown significant improvement in several engineering applications, especially in enhancing the thermal properties.^34–38^

In parallel with the nanofluid investigations, the discovery of carbon nanotubes (CNTs) by Iijima in 1991 (ref. 39) attracted significant attention in the engineering applications, especially in thermal science.^40,41^ CNTs have shown remarkable thermal properties *i.e.*, high thermal conductivity (2000–3000 W m^−1^ K^−1^), which is higher than water by 3000 times.^42–45^ Accordingly, many researchers have studied the thermophysical properties of nanofluids using CNTs.^46–50^ The properties of the nanofluids can be enhanced and modified to meet the application inquires by controlling the nanoparticles properties including; textural properties, morphology and topology and the nanofluids concentrations and operation conditions, taking in consideration the nanofluids' stability.^28,50–53^ Hence, the agglomeration of nanoparticles which destabilize the nanofluids behaviours can affect heat transfer process.

Individual CNT's, polymers or nanoparticles still cannot meet new technologies requirements and to solve the most critical problems in the engineering world. So, researchers start merging CNT, polymers and nanoparticles in order to get higher thermal properties in the same field.^54,55^ To overcome this challenge and get a homogenous solution of nanofluid, different strategies have been implemented such as chemical, physical and mechanical methods.^56–61^

In our previous study,^50^ the enhancement of both specific heat capacity and thermal conductivity of nanofluids free of surfactants using carbon nanotubes chemically functionalized with polyethylene glycol has been investigated. As a continuation of our previous work, this study mainly focused on the thermophysical properties of nanofluids with impregnated copper oxide on the surface of CNTs (CNT–CuO/water nanofluids). The specific heat capacity, thermal conductivity and viscosity of nanofluids were investigated. Additionally, the heat transfer performance of the nanofluids was considered by measuring the heat-transfer rate, pressure drop and pumping power under turbulent flow regimes using shell and tube heat exchanger.

## 2. Materials and experimental work

### 2.1 Materials

In this study, commercial multi-wall carbon nanotubes (CNTs) were purchased from Chengdu Organic Chemicals Co. Ltd. (Times Nano, China). The purity of these CNTs was 95% with diameters varying from 20 to 40 nm, length varied from 10 to 30 μm, and the specific surface area 200 m^2^ g^−1^. Cupric nitrate Cu(NO_3_)_2_·9H_2_O (99% purity) purchased from Sigma and Aldrich Co. Ltd. was used as a precursor to prepare the copper oxide nanoparticles.

### 2.2 Impregnation of CNTs with copper oxide nanoparticles using incipient wetness impregnation (IWI)

Copper oxide (CuO) nanoparticles were prepared from copper nitrate Cu(NO_3_)_2_·9H_2_O precursor using wet impregnation technique. A fixed amount of molar ratio of copper nitrate salts was dissolved in ethanol solution to obtain the targeted weight doping of about 1 and/or 10 wt% of copper oxide (CuO) nanoparticles on the surfaces of CNTs. The impregnation of copper nanoparticles on the surfaces of CNTs was carried out under ultrasonic sonication condition for 30 minutes at a constant temperature of 25 °C. The sonication process is an important step to deagglomerate the clumps of CNTs. Then, the prepared samples (*i.e.*, 1 and 10 wt% CuO)/CNTs composites were dried in a vacuum oven for 12 hours at 60 °C. Eventually, the dried samples were subjected to calcination step at 350 °C for 6 hours to produce copper oxide nanoparticles on the surfaces of carbon materials.

The impregnated carbon nanotubes with copper oxide (CNT–CuO) were characterized using high-resolution transmission electron microscopy (HR-TEM, FEI Talos F200X) and scanning electron microscopy (FE-SEM, Tescan Lyra 3) to study the microstructure and morphology of the nanoparticles. The TEM was performed by applied alcohol to the nanotube film before transferring to a carbon-coated copper grid. The SEM imaging was conducted by varying the magnification in the secondary electron and backscattering mode utilizing an accelerating voltage of 20.0 keV. Thermo-Gravimetric Analysis (TGA, model SDT Q600 TA Instrument) was also performed to quantify the amounts of copper nanoparticles on CNTs by completely oxidized the whole carbon at 700 °C with 10 °C min^−1^ heating rate under air. Additionally, the interaction between the CuO and the surface of CNT was investigated using the X-ray photoelectron spectroscopy (XPS) analysis.

### 2.3 Preparation of water-based nanofluids

The two-step method,^62,63^ the most widely used technique, was used to prepare CNT–CuO/water based nanofluids. The copper oxide nanoparticles decorated on the surface of CNTs (CNT–CuO) were initially prepared using Incipient Wetness Impregnation (IWI) as described before. Then, the CNT–CuO nanoparticles powder were gently dispersed in the distilled water as a base fluid at different weight loadings by applying high ultrasonic waves for 30 minutes using probe sonicator at 100% amplitude. Ultrasonication step was necessary to weaken the van der Waals interactions between the particles, thus disperse them homogeneously in the solution.^28^ The stability of nanofluids was visually observed at different interval times to monitor the suspended particles for a long period of time.

### 2.4 Determination the thermophysical properties of nanofluids

Differential Scanning Calorimetry, (DSC Q1000, model TA Instruments, USA), connected to the rapid cooling system, RCS 90 with nitrogen gas was used to measure the specific heat capacity (*C*_p_) of the prepared nanofluids. The DSC measurements were carried out by heating the nanofluids samples from 25 °C to 50 °C with a heating rate of 1.5 °C min^−1^. Then, the sample was cooled down to the room temperature. Three measurements were taken for each sample of nanofluids, and then these values were averaged to yield the data with 5% standard deviation. The mean value of these measurements was used in the subsequent results.

Moreover, the thermal conductivity (*k*) of nanofluids was measured using Mathis TCi system (Mathis Instruments Ltd.) at different concentrations (0.01, 0.05 and 0.1 wt%) of 1 and 10 wt% CNT–CuO and a constant temperature of 35 °C.

In addition, the dynamic viscosity of the nanofluids was measured using a Stormer viscometer (Thomas Scientific, USA). The viscosity was estimated by measuring the time needed for the inner cylinder to perform 100 revolutions in response to motivating weight. The measurements were taken at temperatures ranging from 25 to 65 °C for different mass concentrations of nanofluids.

### 2.5 Heat transfer system

**Fig. 1:**
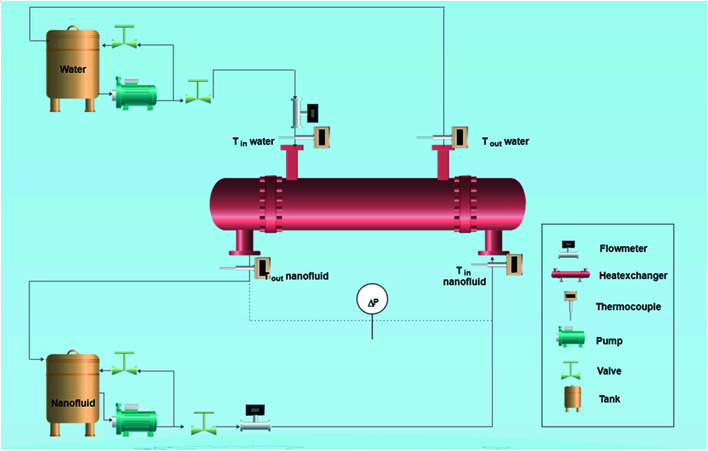
A schematic diagram of the shell and tube heat exchanger system (not scaling).

A fully PC-controlled tube and shell heat exchanger system, as shown in Fig. 1, was used to measure the heat-transfer rate of nanofluids under different flow conditions. The nanofluid was pumped through the tube-side which consists of stainless steel-type SS 316Ti with 2980 mm length (*L*), 10 mm inside diameter (ID) and 14 mm outside diameter (OD). Distilled water was fed through the shell-side which is made of a borosilicate glass 3.3 with 30 mm diameter. Four thermocouple temperature sensors (Methermocouples (Fe–CuNi-type)) were inserted in the shell and the tube sides to measure the inlet and outlet temperatures of each stream flow. Another thermocouple was inserted into the nanofluid vessel (12 litre capacity) to measure the variations in the temperature. The pressure drop of the flowing fluid in both tube and shell sides was measured using two differential pressure transmitters. The heat transfer experiments were performed by changing the mass flow rate of nanofluids from 200 kg h^−1^ to 640 kg h^−1^ and keeping the temperature difference between the inlet tube and shell (Δ*T*) fixed at 10 °C. Therefore, under these flow conditions, the system is ensured to be under turbulent flow region. Then, the inlet and outlet temperatures of both tube and shell and the pressure drop of tube side were measured, after reaching the steady state.

## 3. Results and discussion

### 3.1 Surface characterization of raw and modified CNTs

**Fig. 2:**
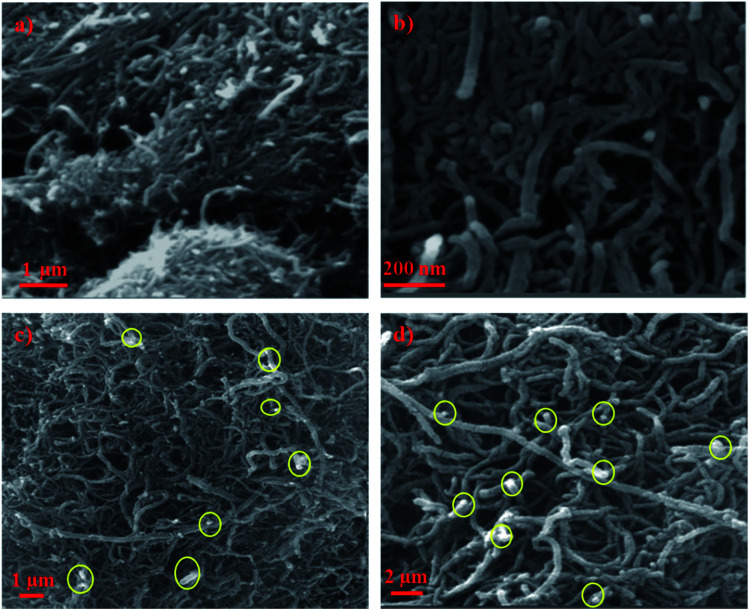
FE-SEM images of (a) CNT low magnification (b) CNT high magnification (c) CNT–1 wt% CuO and (d) CNT–10 wt% CuO.

The FE-SEM micrographs at low and high magnifications of raw and impregnated CNTs are displayed in Fig. 2. It shows that the pristine CNTs are randomly oriented and agglomerated like cotton clumps. The high magnification image (Fig. 2b) displays the characteristic features of CNTs where the diameter ranging from 20 to 40 nm. However, Fig. 2c and d, show the SEM images of doped CNTs with copper oxide nanoparticles in yellow circles at 1 wt% and 10 wt% loadings, respectively. The presence of copper oxide nanoparticles on the surfaces of the CNTs, appearing as white spots, are clearly observed which formed white crystal structures of CuO nanoparticles with small sizes and irregular shapes.

**Fig. 3:**
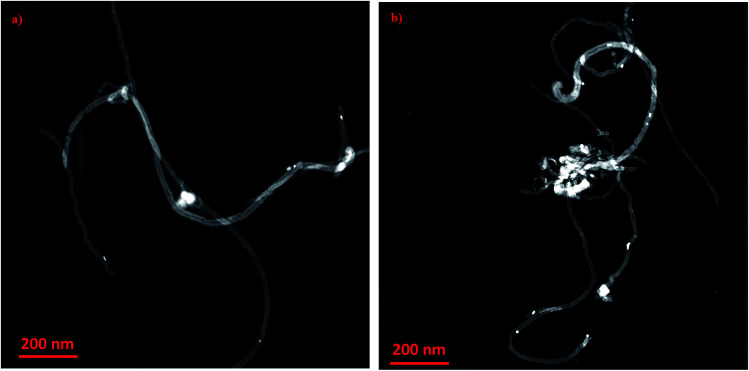
HR-TEM images of copper oxide nanoparticles impregnated on CNT at different loadings (a) 1 wt% CuO and (b) 10 wt% CuO.

Fig. 3 depicts micrographs of CNTs doped with copper 1 wt% and 10% copper oxide nanoparticles. From the figures, the size of nanoparticles appeared to be in the range of 8–12 nm. It can be observed that, when CuO loading is increased, nanoparticles tend to agglomerate and form the clusters as it can be seen in Fig. 3b. No major changes on the surfaces of doped CNTs can be seen after calcination and impregnating with CuO nanoparticles.

**Fig. 4:**
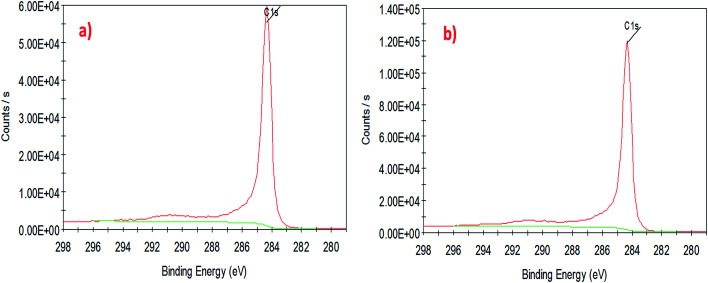
High-resolution XPS spectra of the deconvoluted C 1s peak (a) 1 wt% CNT–CuO, (b) 10 wt% CNT–CuO.

**Fig. 5:**
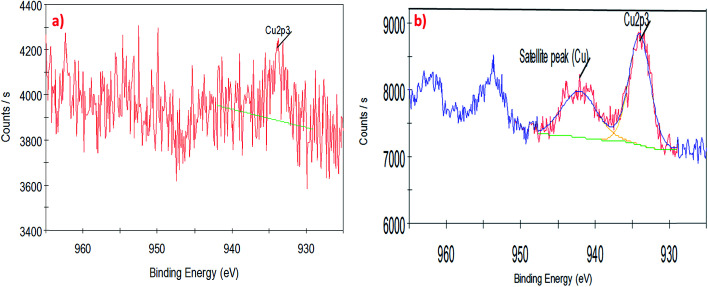
High-resolution XPS spectra of the deconvoluted copper peak (a) 1 wt% CNT–CuO, (b) 10 wt% CNT–CuO.

**Fig. 6:**
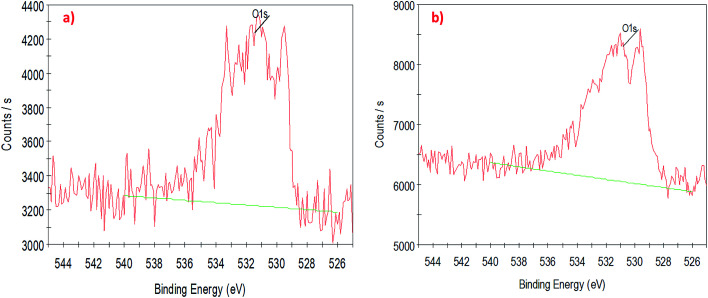
High-resolution XPS spectra of the deconvoluted O 1s peak (a) 1 wt% CNT–CuO, (b) 10 wt% CNT–CuO.

To understand the interaction of CuO nanoparticles on the surfaces of CNTs, XPS analysis was employed for the surface analysis of the samples. Fig. 4, 5 and 6 show the deconvoluted C 1s, Cu 2p_3_ and O 1s spectra of nanocomposite at different loadings of copper oxide. The XPS patterns of the resulting materials show a significant Cu 2p_3_ and O 1s signals which characteristic of copper oxide (Fig. 5 and 6) and the C 1s signal characteristic of carbon nanotubes (Fig. 4a and b). It clear from Fig. 4a and b that the deconvolution of C 1s signal is performed through centering the peak at the binding energy (284.33 eV) for both samples.^64^Fig. 5 confirms the presence of copper in the surface of CNT. The signal at 933.83 eV in both samples (*i.e.*, 1 and 10 wt% CuO) attributed to the copper as Cu 2p_3_. However, another important signal of copper was observed at 942.08 eV attributed to satellite peak (Cu) for 10 wt% CuO–CNT (Fig. 5b).^65,66^ This latter peak and the one at 933.83 confirm that the CuO is higher in 10 wt% CuO sample compared with 1 wt% CuO which is also evidenced in Fig. 6a and b for the highest contribution of O 1s at 531.4 eV in 10 wt% CuO–CNT. Therefore, it can be concluded that the XPS patterns verify the presence of copper oxide in the nanocomposite materials which has been obtained by incipient wetness impregnation method.

### 3.2 Thermal degradation analysis of nanoparticles

**Fig. 7:**
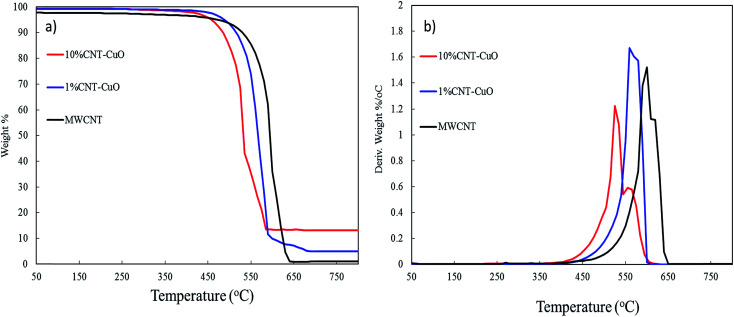
Thermogravimetric analysis (TGA) of CNT and impregnated CNT with copper oxide (a) the weight loss and (b) thermogravimetric (DTG).

Thermogravimetric analysis (TGA) and derivative thermogravimetric (DTG) were used to determine the weight loading of nanoparticles on the surfaces of CNTs and measured their thermal stability. For this purpose, the TGA-DTG analysis was conducted under air condition for the range of temperatures from 25 °C to 750 °C under a heating rate of 10 °C min^−1^. Fig. 7a and b shows the oxidation of the pristine CNT started at 450 °C, while the highest thermal oxidation rate was found at 600 °C as confirmed by derivative weight loss (Fig. 7b). The complete oxidation of the CNTs with zero residual was observed at 670 °C. However, the oxidation region of the CNTs impregnated with 1 wt% copper nanoparticles started at 500 °C and maximum weight loss reached at 550 °C. Moreover, by increasing the loading of CuO to 10 wt%, the oxidation peak shifted to the lower temperature in which initial oxidation at this condition started at 450 °C with maximum weight losses at 500 °C and complete oxidation at 540 °C. The TGA results confirm the presence of the nanoparticles on the CNT surface which is in good agreement with results of TEM, SEM and XPS. The CuO plays a significant catalytic role in oxidizing the CNT. Moreover, the fast oxidation of doped CNTs compared to undoped CNTs is due to heat localization of metal nanoparticles which accelerate the heat transfer and increase the oxidation rate.^28^

### 3.3 Viscosity of nanofluids

**Fig. 8:**
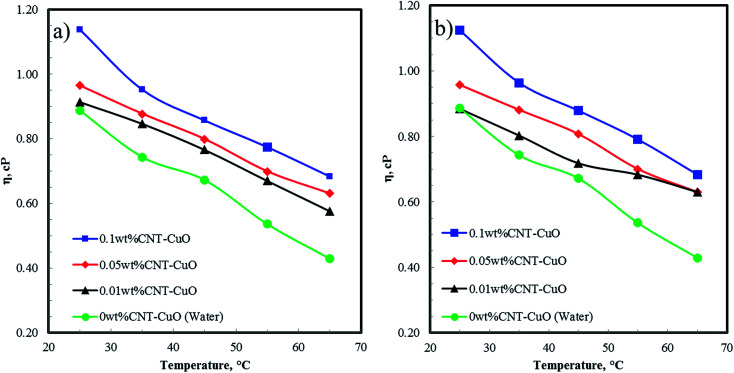
The viscosity of nanofluids at a different concentrations with respect to temperature for (a) CNT–1 wt% CuO, and (b) CNT–10 wt% CuO.

Fig. 8a and b shows the viscosity of nanofluids as a function of temperature at different CNTs concentrations of undoped and doped with 1 and 10 wt% of copper oxide nanoparticles. The results showed that the viscosity of nanofluids slightly increases from 2 to 5% increment at the low concentration of CNTs at 0.01 and 0.05 wt%, while a remarkable increase ∼22% was observed at a high concentration of nanoparticles at 0.1 wt%. The increase in the viscosity is attributed to the higher suspension concentration of the nanoparticles in water, which cause higher internal viscous shear stresses.^67–69^ Interestingly, there was no significant change in the viscosity of nanofluids when loading copper oxide nanoparticles are increased from 1 to 10 wt% CuO. However, the viscosity of nanofluids decreases significantly with an increase in fluids temperature. This can be explained by the fact that the elevated temperature weakens the intermolecular forces of both nanoparticles and fluid in the suspension and increase their kinetic energy.^50^

### 3.4 The thermal properties of nanofluids

**Fig. 9:**
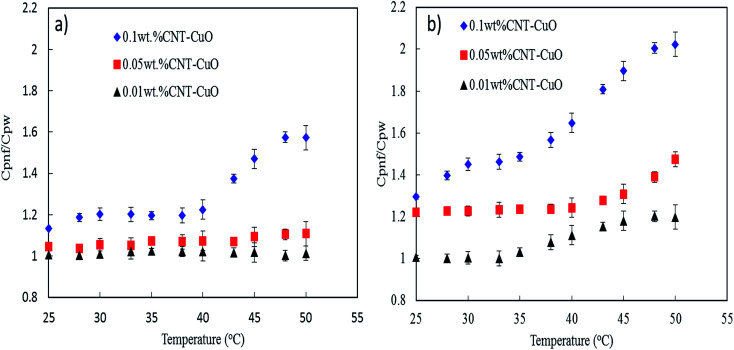
Specific heat capacity of nanofluids with respect to temperature (a) CNT–1 wt% CuO, (b) CNT–10 wt% CuO.

The specific heat capacity of the prepared nanofluids with different concentrations of undoped and doped CNTs was measured by DSC technique. Fig. 9a and b reveals the enhancement of the heat capacity of the nanofluids (*C*_p,nf_/*C*_p,w_) as a function of temperature, where *C*_p,nf_ and *C*_p,w_ are the specific heat capacity of nanofluids and water, respectively. As shown in Fig. 9, the heat capacity of nanofluids significantly increases with an increase in the concentration of both undoped and doped CNTs. The doped CNTs were shown to have higher heat capacities enhancement compare to undoped CNTs. The maximum enhancement of the specific heat capacities of the doped CNTs with 1 and 10 wt% CuO at 0.1 wt% were found to be 60% and 102% higher than that of the pure water, respectively. Consequently, this enhancement in heat capacity was higher by 81% compared with undoped CNT-nanofluids as reported in our previous work, where the *C*_p_ value was 4.91 J kg^−1^ K^−1^ under similar conditions.^28^ The unusual enhancement in the specific heat capacity of nanofluids could be explained based on our proposed mechanisms in the previous works.^28,50^ Accordingly, the first mechanism depends on the dispersion of nanoparticles which acting as the heat sink between the CNTs networks. This phenomenon was satisfied by modifying the surface of CNT physically with hydrophilic materials and mechanically by applying the ultrasonic sonication waves to break down the van der Waals interaction forces, thus enhancing the nanoparticles dispersion in water.^45,50^ The second mechanism basically relates to the interfacial interactions forces between the nanoparticles and water which play a major role in changing the water characteristic, thus altering its thermal properties. The third mechanism corresponds to the unique thermal properties of nanoparticles which allowing the nanofluid to store the heat faster than water and reaching the maximum capacity within a shorter time and evenly distributing the heat in the water.

**Fig. 10:**
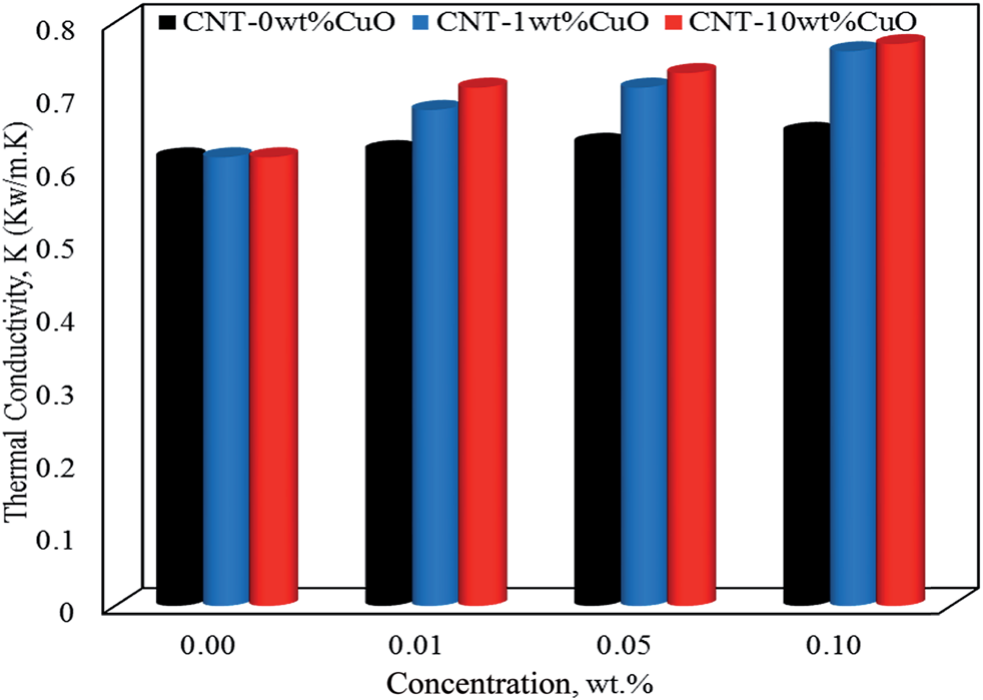
The thermal conductivity of nanofluids at 35 °C for pure CNT, CNT–1 wt% CuO and CNT–10 wt% CuO.

In terms of thermal conductivity of nanofluids, Fig. 10 shows thermal conductivity values of nanofluids at 35 °C with different concentrations of 0.01, 0.05 and 0.1 wt% for pure and impregnated CNT. The thermal conductivity of the water base fluids was remarkably improved by dispersing the undoped and doped CNTs. Worth noting that the undoped CNTs enhanced the thermal conductivity of the nanofluids by 6% while with 1 and 10 wt% CuO nanoparticles doped on CNTs a tremendous increase by 23% and 26%, respectively were observed at 0.1 wt% nanofluid concentration. Interestingly, the thermal conductivity results obtained using our nanoparticles (CNT–CuO) are higher than that of CuO nanoparticles as reported in the literature. For instance, Lee *et al.*,^70^ showed an enhancement of about 20% in thermal conductivity at 4 vol% of CuO nanoparticles in ethylene glycol mixture while Manimaran *et al.*,^27^ found that the thermal conductivity of CuO/water-based nanofluids is increased to 12.4% compared with water.

Indeed, the mechanisms of enhancement in thermal conductivity of nanofluids have been addressed by different research groups.^4,58,68,71^ These suggested mechanisms mostly depend on the nanoparticles dispersion,^72,73^ size and shape of nanoparticles^23,74^ and Brownian motion.^51,75^ Based on that, we hypothesise that the CNT–CuO nanoparticles are believed to obey those factors to explain the enhancements on the thermal enhancements of the nanofluids. The most significant and dominant factor is the stability/dispersion of nanofluids (nanoparticles disaggregation) which was achieved here by either physical or mechanical approaches. For instance, the mechanical method (a short-term effect) was achieved by applying ultrasonic sonication waves to break down the van der Waals interaction forces while the physical approach (a long-term effect) was obtained by modifying the surfaces of the CNTs with nanoparticles (CuO) which reduce their aggregation and enhanced their dispersion into the solution. Another factor is related to the particles size effects, which represents as the ratio of surface area to the volume (*A*/*V*). Hence, the particles in nanosize have (*A*/*V*) ratio higher than the one in the bulk or micro sizes. Thus, the average particle size of copper oxide nanoparticles doped on CNTs is ∼10 nm (Fig. 3), where the ratio (*A*/*V*) is 1000 times greater than a material with 10 μm diameter. This means that the suspended nanoparticles have higher ability to store and transfer energy *via* heat capacity and thermal conductivity than fluids containing coarse solid particles on a micron scale. Another mechanism due to the cylindrical shape of the liquid molecule layers which behave like a semi-solid layer on the surfaces of nanoparticles.^50^ Thus, high thermal properties were found for nanoparticles that have a cylindrical shape as a rapid heat transfer along a relative distance in cylindrical particles is obtained.^75^ In addition, another mechanism can be observed due to the interfacial interactions forces between the nanoparticles and the base fluids. This could affect the mobility of nanoparticles, due to the Brownian motion, and may act as “agitators” to encourage thermal transport and enhance the effective heat capacity in nanofluids.^13,28,76^

### 3.5 Heat transfer of nanofluids

The heat-transfer rate of the prepared undoped and doped CNTs with 1 and 10 wt% CuO nanofluids were experimentally investigated at different concentrations of 0.01, 0.05 and 0.1 wt% using shell and tube heat exchanger. The inlet temperature of the nanofluids in tube-side was fixed at 35 °C using heating bath while the inlet temperature of water in the shell-side was fixed at 20 °C. Additionally, the flow rate of nanofluids was ranged from 200 to 640 kg h^−1^.

**Fig. 11:**
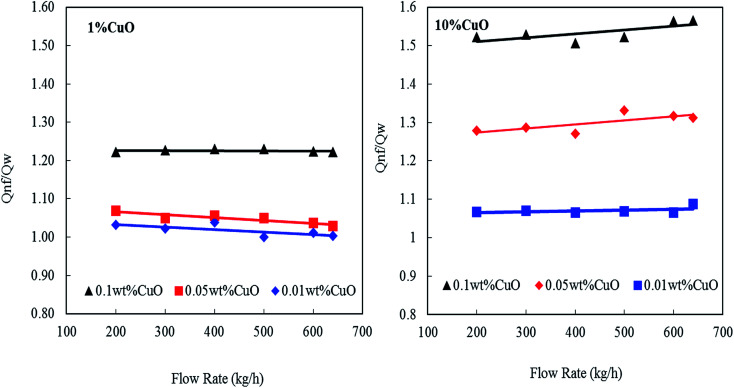
Enhancement heat transfer of nanofluids at different concentration of nanoparticles and flow rates.

Fig. 11a and b shows the effect of nanofluids concentrations (0.01, 0.05 and 0.1 wt% CNT–CuO) on the enhancement of the heat transfers at a different nanofluids flow rate. The heat-transfer rate in the tube-side was calculated based on the following equation:

1 *Q* = *m*_n_*C*_p,nf_(*T*_n,in_ − *T*_n,out_)

where *Q* is the heat-transfer rate in the tube, *m*_n_ is the mass flow rate for nanofluids, *C*_p,nf_ is the specific heat capacity of nanofluids, *T*_n,in_ is the tube-inlet temperature and *T*_n,out_ is the-tube outlet temperature.

It can be observed that the heat transfer ratio 
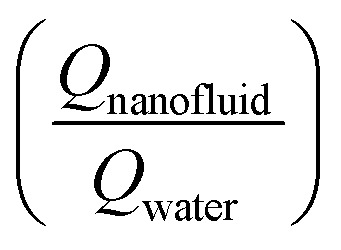
 is significantly increased with the increase in the concentration of nanoparticles. Interestingly, by increasing the loading of the copper oxide nanoparticles on the surfaces of CNTs from 1 to 10 wt% the heat-transfer rate is dramatically increased from 23% to 58% at 0.1 wt% CNT–CuO compared with water at the same operating conditions. Also, it can be found that the 10 wt% of CNT–CuO nanocomposite shows better heat transfer compared to 1 wt% loading of CuO at the same flow rate. For instant, at flow rate 600 kg h^−1^ as the 10 wt% of CNT–CuO nanofluid concentration changes from 0.01% to 0.1% the ratio of heat-transfer increases from 1.1 to 1.55 while for the 1 wt% CNT–CuO at the same flow rate the ratio increases from 1 to 1.23.

These improvements in the heat-transfer rate can be attributed to the enormous increase in heat capacities and thermal conductivity of the CNTs/copper oxide. Worth noting here that a good dispersion and stability of the doped CNTs were observed leading to the high enhancement in heat transfer of nanofluids (*i.e.*, *Q*). In other words, the heat transfer improvement of nanofluids was higher than the corresponding value of heat capacity itself.^19^ Several reasons could be behind this observation; firstly, the measurements of specific heat capacity were taken under a static condition, whereas the measurement of heat-transfer rate (*Q*) was conducted in dynamic flow condition. Secondly, the interactions between of the flowing fluid and the nanoparticles might be another reason for the substantial rise in heat transfer rate. Thirdly, another possible mechanism relates to surface phenomena where a small fine-layer of the nanoparticles was deposited on the inner wall of the steel tube, and thus enhance the thermal conductivity.^28^

**Fig. 12:**
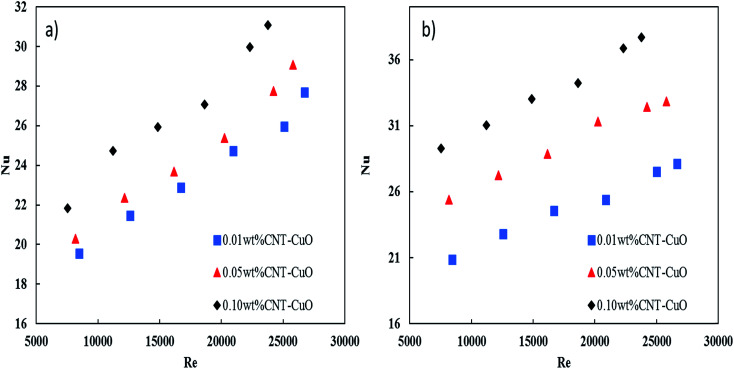
The enhancement of Nusselt's number *versus* Reynold's number at different concentration of (a) 1 wt% CNT–CuO (b) 10 wt% CNT–CuO.

Moreover, a similar trend was observed when the heat transfer was expressed in terms of Nusselt's number (Nu), as shown in Fig. 12. The Nu number was calculated using the following equation;2
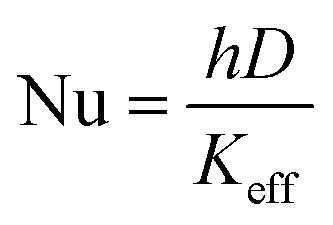
while the heat transfer coefficient (*h*) was expressed as;3
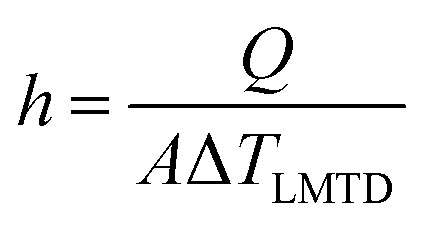
where *K*_eff_ in the effective thermal conductivity of nanofluids, *D* is the inner diameter of the tube and *A* is the surface area of the tube.

The Nu number not only reflects the effect of thermal conductivity of nanofluids but also the effect of specific heat capacity. As shown in Fig. 12, the relation between the two dimensionless numbers (Nu and Re) for the two types of nanofluids is clearly noticed at different concentrations of nanoparticles. It can observe that the Nu number for both nanofluids is significantly enhanced with the increase in both nanoparticles concentration and Re number, which strongly indicates the increase in the convection heat transfer of nanofluids. This significant increase in Nusselt number is attributed to the increase of thermal conductivity and heat capacity of the nanofluids.

Overall, from the experimental results, the heat-transfer rate and the Nusselt number of CNT–CuO/water-based nanofluids are higher than the pure water, and CuO/water-based nanofluids as reported in the literature.

### 3.6 Pressure drop of nanofluids

**Fig. 13:**
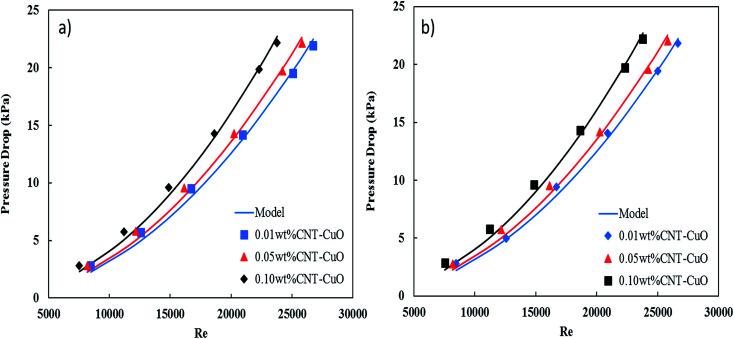
Variation in the pressure drop of nanofluids with respect to the Re in the steel tube for (a) 1 wt% CNT–CuO and (b) 10 wt% CNT–CuO.

The pressure drop of nanofluids along the tube-side was measured using a differential pressure transmitter with an accuracy of ±5 mbar. The pressure drop measurements were considered under the turbulent flow regime where the Reynolds number varying from 5000 to 25 000. Fig. 13a and b shows the variation of the pressure drop with respect to Re for impregnated CNTs with copper oxide nanoparticles. It is clearly observed that the pressure drop of nanofluids increased slightly with an increase in the nanoparticles concentrations for both loadings of copper oxide. Worth noting here that no noticeable changes in the pressure drop were observed when the loading of copper oxide nanoparticles increased to 10 wt%. The change in pressure drop was relatively low (*i.e.*, 5–8%) when it is compared with other proposed nanofluids and commercial fluids.^77^ Nevertheless, the reason for this increase in the pressure drop of nanofluids could be explained by the increase in the viscosity as discussed in Section 3.3. Hence, the viscosity of nanofluids is increased to 14%, 18% and 28% respectively at 0.01%, 0.05% and 0.1% weight concentration of CNT–CuO nanofluids. In comparison, Fotukian *et al.*,^17^ investigated experimentally the convective heat transfer and pressure drop of CuO/water nanofluid under turbulent flow condition in a circular tube. Their results showed that the convective heat transfer increased by 25% while the pressure drop was 20% higher than that of pure water.

Moreover, the pressure drop of nanofluids flowing through the pipe under turbulent flow conditions is not only function with viscosity but also the fluid velocity (*v*^2^) and density (*ρ*), the tube length (*L*) and diameter (*D*), and the friction factor (*f*), which is expressed as4
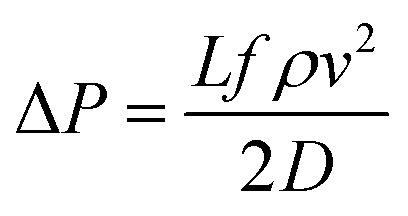


In this correlation (eqn (4)) one parameter was fit, friction factor (*f*), for each flow rate experiment using Mathematica software to get the proper value of *f* with minimum value of stranded error as shown in Table 1.

**Table 1:** The friction factor of the stainless-steel tube through nanofluids flowing

nanofluids, wt%	1 wt% CuO		10 wt% CuO	
	*f*	*χ* ^2^	*f*	*χ* ^2^
0.01	0.029164	3.80 × 10^−4^	0.0294406	4.76 × 10^−4^
0.05	0.029507	4.63 × 10^−4^	0.0296783	4.62 × 10^−4^
0.10	0.029741	4.79 × 10^−4^	0.0297785	4.60 × 10^−4^

Fig. 13 also shows an excellent fitting between the experimental data and eqn (4), and this was indicated by the lowest values of *χ*^2^. It can be noted from the friction factor values that there is no noticeable increase in the friction factor with nanoparticles concentration. Nevertheless, a small amount of the nanoparticles (CNT/CuO) deposition was observed on the inner-tube surface which might have a significant effect on the pressure drop especially at a high concentration of the nanoparticle. Same phenomenon and trends have been observed in our previous works.^28,50^

### 3.7 Pumping power of nanofluids

As discussed early, a remarkable increase in the heat transfer of nanofluids with increasing nanoparticles concentration was noticed using modified CNT with CuO. This enhancement was associated with a very slight increase in the pressure drop along with the tube-side of the heat exchanger. However, taking the pumping power into consideration would lead to evaluate the nanofluid efficiency with a better performance especially at high concentrations. From the energy savings point of view, the addition of nanoparticles into a water will not only enhance heat transfer but also will increase the pressure drop. Therefore, the enhancement of heat transfer should be synchronized with minimum pressure drop in order to optimize and design the heat exchanger with minimal pumping power.

**Fig. 14:**
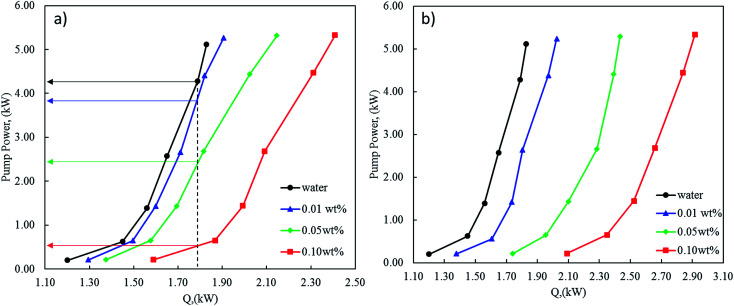
Pump power required as a function of the heat generated at different concentrations of nanofluids (a) 1 wt% CNT–CuO (b) 10 wt% CNT–CuO.

In this regard, Fig. 14 shows the relation between the heat-transfer rate (*Q*) and the pumping power (PP). The pumping power increases with heat flow and nanofluids concentrations. Indeed, for a given heat flow, pumping power increases considerably with the nanofluid concentrations. For example, in the case of 0.1 wt% of nanofluid with 1 wt% CNT–CuO, to exchange 1.8 kW approximately 500 W is required which is 9 times lower than the power (4.3 kW) required to exchange the same amount of heat using water (Fig. 14a). In the same manner, for the case of 0.1 wt% of nanofluid with 10 wt% CNT–CuO (Fig. 14b), whereas the pump power is approximately 5 times less than the pure water at low concentration due to the sharp increase in heat transfer compared with the minimal pumping penalty as discussed earlier. These promising findings would increase the interest on such materials leading to a high and immediate impact on thermal management devices and equipment.

## 4. Conclusion

The heat-transfer performance and pressure drop of nanofluids through a horizontal tube of the heat exchanger under the turbulent flow condition were successfully investigated. The surface of CNTs was modified by impregnating different loadings of copper oxides nanoparticles on the CNT surface. The CNT–CuO nanocomposites were characterized by TEM, XPS, SEM and TGA. The characterization results of nanoparticles confirmed that the surface of CNTs was successfully doped with copper oxide nanoparticles at 1 and 10 wt% loading. The two-step method was used to prepare the nanofluids by dispersing the modified CNTs with copper oxide nanoparticles into the water. The thermal–physical properties (specific heat capacity, thermal conductivity and viscosity) of the prepared nanofluids were measured using DSC, Mathis TCi and Stormer viscometer. The specific heat capacity results of nanofluids were significantly enhanced with the increase of the nanoparticles concentration. The maximum enhancement of the specific heat capacities of the doped CNTs with 1 and 10 wt% CuO at 0.1 wt% were 60% and 102% higher than that of pure water, respectively. The heat-transfer rate of nanofluid showed a significant increase in the presence of CNTs/CuO nanoparticles. Interestingly, the pressure drop of the heat exchanger system using our nanofluid was insignificantly affected by the increase in nanoparticles concentration, therefore the heat exchangers can be designed with low pumping power.

## Conflicts of interest

There are no conflicts to declare.

## Supplementary Material
